# Th1 type lymphocyte reactivity to metals in patients with total hip arthroplasty

**DOI:** 10.1186/1749-799X-3-6

**Published:** 2008-02-13

**Authors:** Nadim James Hallab, Marco Caicedo, Alison Finnegan, Joshua J Jacobs

**Affiliations:** 1 Department of Orthopedic Surgery, Rush University Medical Center, Chicago, IL 60612, USA; 2 Department of Rheumatology, Rush University Medical Center, Chicago, IL 60612, USA

## Abstract

**Background:**

All prostheses with metallic components release metal debris that can potentially activate the immune system. However, implant-related metal hyper-reactivity has not been well characterized. In this study, we hypothesized that adaptive immunity reaction(s), particularly T-helper type 1 (Th1) responses, will be dominant in any metal-reactivity responses of patients with total joint replacements (TJAs). We tested this hypothesis by evaluating lymphocyte reactivity to metal "ions" in subjects with and without total hip replacements, using proliferation assays and cytokine analysis.

**Methods:**

Lymphocytes from young healthy individuals without an implant or a history of metal allergy (Group 1: n = 8) were used to assess lymphocyte responses to metal challenge agents. In addition, individuals (Group 2: n = 15) with well functioning total hip arthroplasties (average Harris Hip Score = 91, average time in-situ 158 months) were studied. Age matched controls with no implants were also used for comparison (Group 3, n = 8, 4 male, 4 female average age 70, range 49–80). Group 1 subjects' lymphocyte proliferation response to Aluminum^+3^, Cobalt^+2^, Chromium^+3^, Copper^+2^, Iron^+3^, Molybdenum^+5^, Manganeese^+2^, Nickel^+2^, Vanadium^+3 ^and Sodium^+2 ^chloride solutions at a variety of concentrations (0.0, 0.05, 0.1, 0.5, 1.0 and 10.0 mM) was studied to establish toxicity thresholds. Mononuclear cells from Group 2 and 3 subjects were challenged with 0.1 mM CrCl_3_, 0.1 mM NiCl_2_, 0.1 mM CoCl_2 _and approx. 0.001 mM titanium and the reactions measured with proliferation assays and cytokine analysis to determine T-cell subtype prominence.

**Results:**

Primary lymphocytes from patients with well functioning total hip replacements demonstrated a higher incidence and greater magnitude of reactivity to chromium than young healthy controls (p < 0.03). Of the 15 metal ion-challenged subjects with well functioning total hip arthroplasties, 7 demonstrated a proliferative response to Chromium, Nickel, Cobalt and/or Titanium (as defined by a statistically significant >2 fold stimulation index response, p < 0.05) and were designated as metal-reactive. Metals such as Cobalt, Copper, Manganese, and Vanadium were toxic at concentrations as low as 0.5 mM while other metals, such as Aluminum, Chromium, Iron, Molybdenum, and Nickel, became toxic at much higher concentrations (>10 mM). The differential secretion of signature T-cell subsets' cytokines (Th1 and Th2 lymphocytes releasing IFN-gamma and IL-4, respectively) between those total hip arthroplasty subjects which demonstrated metal-reactivity and those that did not, indicated a Th1 type (IFN-gamma) pro-inflammatory response.

**Conclusion:**

Elevated proliferation and production of IFN-gamma to metals in hip arthroplasty subjects' lymphocytes indicates that a Th1 (vs. Th2) type response is likely associated with any metal induced reactivity. The involvement of an elevated and specific lymphocyte response suggests an *adaptive *(macrophage recruiting) immunity response to metallic implant debris rather than an *innate *(nonspecific) immune response.

## Background

Implant related metal hypersensitivity remains a relatively unpredictable and poorly understood phenomenon. [1-3] Dermal hypersensitivity to metal is common, affecting about 10–15% of the population. [1,2,4,5] Dermal contact and ingestion of metals have been reported to cause immune reactions which most typically manifest as skin hives, eczema, redness and itching. [1,5-7] Although little is known about the short and long term pharmacodynamics and bioavailability of circulating metal degradation products in vivo,[4,8,10] there are many case and group studies documenting metal reactivity responses temporally associated with implantation of metal components.

All metals in contact with biological systems corrode[11,12] and the released metal ions, while not sensitizers on their own, can act as haptens, activating the immune system by forming complexes with native proteins. [4,13,14] Metal-protein complexes are considered to be candidate antigens for eliciting hypersensitivity responses. Metals known as sensitizers (haptenic moieties in antigens) are beryllium,[15] nickel,[5-7,15] cobalt[15] and chromium[15] while occasional responses have been reported to tantalum,[16] titanium [17,18] and vanadium. [16] Nickel is the most common metal sensitizer in humans followed by cobalt and chromium. [1,4-7]

The specific T-cell subpopulations associated with metal hypersensitivity, the cellular mechanism of recognition/activation, and the antigenic metal-protein determinants created by implant metals remain incompletely characterized. Th1 (T-helper type 1) and Th2 (T-helper type 2) correspond to CD4+ αβ TCR T cell subsets that provide help to cells of both the innate and adaptive immune systems. We hypothesized that adaptive Th1 mediated responses will be the more prevalent type of hypersensitivity response to metal in patients with total hip arthroplasty (THA). We investigated this hypothesis by evaluating the reactivity of peripheral blood lymphocytes to metal challenge agents (Cobalt, Chromium, Nickel and Titanium) in subjects with and without hip arthroplasties using proliferation and cytokine assays. We studied lymphocyte responses in three different subject groups: i) young healthy individuals without implants and without a history of metal allergy were used to assess general lymphocyte reactivity to various concentrations of metal challenge agents; ii) individuals with hip arthroplasty were determined to be either metal-reactive or metal-nonreactive by cell proliferation studies, and iii) age matched controls without implants were used to assess lymphocyte subtype dominance by cytokine release profiles.

## Materials and methods

Contrasting the metal reactivity responses of healthy subjects without implants and subjects with implants was used to assess the immunogenic effects of metals found retrospectively in orthopaedic implant cohorts (n = 31 total subjects). Informed consent was obtained from all subjects after Institutional Review Board review and approval. Group 1 consisted of young healthy subjects without any metallic implants (n = 8, 4 male and 4 female subjects with an average age of 29, range 23 to 36) and was primarily used for determination of relative metal toxicity and upper limits of challenge dose. In Group 1 subjects lymphocyte proliferation in response to a variety of metals (Aluminum-Al^+3^, Cobalt-Co^+2^, Chromium-Cr^+3^, Copper-Cu^+2^, Iron-Fe^+3^, Molybdenum-Mo^+5^, Manganeese-Mn^+2^, Nickel-Ni^+2^, Vanadium-V^+3 ^and Sodium-Na^+2 ^chloride solutions, Sigma, St Louis, MO) at a variety of concentrations (0.0, 0.05, 0.1, 0.5, 1.0 and 10.0 mM) was studied to establish toxicity thresholds. Group 2 consisted of 15 hip arthroplasty single-implant type subjects with well functioning (average Harris Hip Score = 91) total hip arthroplasties of the same design (Harris-Galante, Zimmer, Warsaw, IN) with 7 males and 8 females (average age 69 yrs, range 55–80 yrs), and an average hip arthroplasty time in-situ of 158 months (range 117–174 months). This implant system is comprised of a Titanium-6%Aluminum-4%Vanadium alloy stem with a Cobalt-Chromium-Molybdenum femoral head, an Ultrahigh Molecular Weight Polyethylene (UHMWPE) acetabular cup in a Titanium-6%Aluminum-4%Vanadium alloy lining. A sample size of 14 in each group will have an 80% power to detect a probability of 0.81 that an observation in one group is less than an observation in the other group using a two-sided Mann-Whitney test with a 0.05 significance level. Two of 15 reported a history of metal allergy and 5 of 15 had moderate osteolysis (proximal focal lesions in excess of 0.5 cm^2 ^in total area on an anteroposterior radiograph and/or distal [diaphyseal] focal lesions greater than 1 cm^2 ^in total area on an anteroposterior radiograph were correlated with the magnitude of articular surface wear). [19] Group 3 subjects were age-matched controls (to Group 2) with no implants (Group 3, n = 8, 4 male, 4 female, average age 70, range 49 to 80). Group 2 subjects with hip arthroplasty were evaluated for lymphocyte reactivity to metals using lymphocyte transformation testing (proliferation assays), and IFN-gamma, IL-2, and IL-4 cytokine analysis. The metal challenge agents for Group 2 subjects were a subset of the more prevalent implant-alloy metals used for appropriate dose determination in Group 1 and consisted of 0.1 mM CrCl_3_, 0.1 mM NiCl_2_, 0.1 mM CoCl_2 _(Sigma, St. Louis, MO) and approx. 0.001 mM titanium (using Titanium saturated culture medium produced through incubation with Titanium beads). Despite its prominence as an implant alloy, titanium could only be tested at such low concentrations because of the insoluble nature of titanium at physiologic pH and its subsequent inability to form ions in solution.

### Proliferation assays

(Lymphocyte Transformation Tests): Serum and Peripheral Blood Mononuclear Cells (PBMCs) were obtained from mononuclear cell fractionation of blood obtained by peripheral venipuncture after obtaining informed consent and Institutional Review Board approval. Peripheral blood mononuclear cells were isolated from 30 mL of peripheral blood using density gradient separation (Ficoll-isopaque, Pharmacia, Piscataway, NJ). Ficoll separated mononuclear cells are generally comprised of 85–95% lymphocytes with 5–13% monocytes and <0.1% dendritic cells with limited contamination (i.e. <5% erythrocytes and <3 granulocytes). Among mixed peripheral blood mononuclear cells populations, lymphocytes are the only cells capable of competitively significant in vitro proliferation upon challenge with an allergen. Thus we used standard Lymphocyte Transformation Testing (LLT) protocol of mononuclear cells to measure lymphocyte proliferation and cytokine production (15–30 × 10^6 ^cells per subject). [20,21] Lymphocytes in all assays were incubated with Dulbeccos Modified Eagles Medium and 10% autologous serum with either no metal (plain media) as a negative control, 0.01 mg/ml phytohemagglutinin (PHA) as a positive control, or metal. Each metal challenge treatment was conducted in quadruplicate (4 wells/treatment). [^3^H]-thymidine was added (1 mCi/culture well) during the last 12 hours of incubation after 5 1/2 days of treatment. At day six, [^3^H]-thymidine uptake was measured using liquid scintillation. Incorporated radioactivity was measured using liquid scintillation Beta plate analysis (Wollac Gatesburg, MD). The amount of [3H]-thymidine incorporation for each metal treatment was normalized to that of the nontreated control producing a ratio, referred to here as the proliferation index or stimulation index, SI.

The SI was calculated using measured radiation counts per minute (cpm):

Stimulation Index = (mean cpm with treatment)/(mean cpm without treatment).

Six days of incubation were used to reproduce, in vitro, the time lag associated with in vivo lymphocyte proliferation in a delayed type hypersensitivity (DTH) response. [20,21]

### Measurement of cytokines in culture media

Cell surface markers have been proposed to differentiate Th1 vs. Th2 subtypes (e.g. Th1 cells have been reported to express both components of IL-12 receptor chains (b1 and b2) while Th2 cells exhibit only IL-12Rb1). [22] However, these methods remain technically problematic and not well established for large scale multi-challenge agent and human cohort characterization. Thus current investigative methods of Th1/Th2 subtypes largely remain the differentiation of signature cytokine expression, i.e. Th1 = Interferon gamma (IFN-gamma), tumor necrosis factor beta (TNF-b) and interleukin 2 (IL-2), and Th2 = Interleukins 4, 5, 6, and 13 (IL-4, 5, 6, and 13). [22-24] Cytokine concentrations in supernatants of lymphocytes cultures (>1 × 10^6 ^PBMCs per well per metal in 48-well plates) were obtained after 48 hrs of incubation with metal challenge agents and were measured by sandwich enzyme-linked immunosorbent assays (ELISA) in 96-well microtitration plates following the manufacturer's instructions. ELISA kits (R&D Systems, Minneapolis, MN) for IL-2, IL-4 and IFN-gamma (assay range from 0.5 to 32 pg/ml) were conducted in triplicate for each concentration of each metal treatment.

### Statistical analysis

Normally distributed data were subjected to statistical analysis using Student's t-tests. Student's t-tests for independent samples with unequal or equal variances were used to test equality of the mean values at a 95% confidence interval (p < 0.05). A sample size of 14 in each group will have an 80% power to detect a probability of 0.81 that an observation in one group is less than an observation in the other group using a two-sided Mann-Whitney test with a 0.05 significance level. Comparisons between groups were limited to comparison of reactivity at each metal concentration. All treatment specific reactivity SI measurements were assumed to be normally distributed. The results from the cytokine assays were not normally distributed as some measurements were below the detection limit. By convention, to calculate group means, cytokine concentrations below the detection limit were assigned a value of one-half the method detection limit. Intergroup comparisons, independent of these means, were made using Kruskall-Wallis non-parametric analysis of variance. The Wilcoxon-Mann-Whitney test was then used if the Kruskall-Wallis test revealed significant differences at p < 0.05.

## Results

### Lymphocyte Proliferation

**Figure 1:**
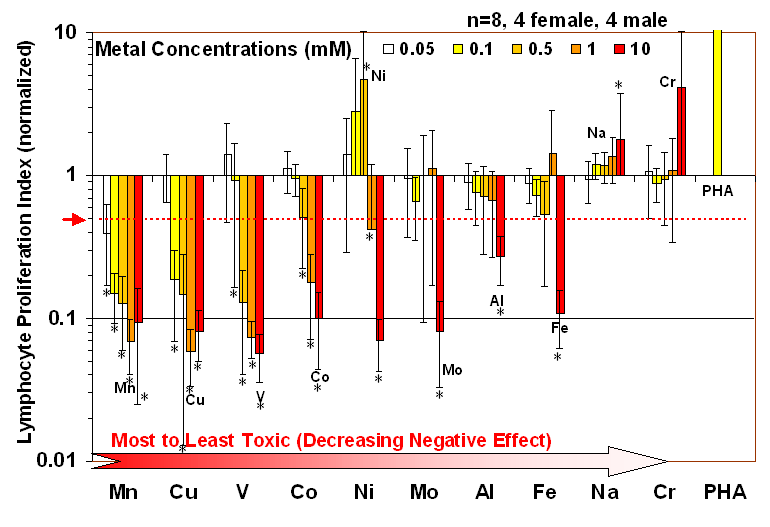
**Human PBMC/lymphocyte proliferation responses to various concentrations of soluble metal challenge are indicated.** Only Nickel indicated significant increases (p < 0.05, t-test) at concentrations of 0.5 and 10 mM. The ranking of the most toxic/inhibitory metals was determined by the lowest concentration of metal required to produce a >50% decrease in the proliferation rate when compared to controls (i.e. <0.5 Proliferation Index). Note: * = p < 0.05, t-test.

The concentration dependent effects of metals on healthy individuals without implants (Group 1) demonstrate the differential effects of implant metals on lymphocytes and, more importantly, verify that the metal dose chosen for Group 2 analysis was non-toxic, i.e. 0.1 mM (Figure 1). Generally, there was a decrease in proliferation associated with an increase in metal concentration for all the metals tested. Only Nickel was found to induce a statistical increase in proliferation at concentrations of 0.5 and 10 mM, respectively (p < 0.05, t-test). Almost all metals induced toxic/inhibitory effects. The most to least inhibitory metals were Manganeese^+2^, Copper^+2^, Vanadium^+3^, Cobalt^+2^, Nickel^+2^, Molybdenum^+5^, Aluminum^+3^, Iron^+3^, and Chromium^+3 ^based on the smallest metal concentrations required to produce a decrease in proliferation of over 50%. The only metals that did not demonstrate a toxicity response at concentrations as high as 10 mM were Chromium and Sodium. Sodium was used as a control to account for the effects of chloride. The most toxic of the metals, Manganeese, Copper and Vanadium, decreased proliferation to below 50% at concentrations below 0.1 mM, indicating that for these particular metals lower concentrations may be appropriate for general metal-reactivity testing using lymphocyte transformation testing. Among these more toxic metals only Vanadium is used in current implant alloys to any significant extent, i.e. Ti-6%Al-4%V. However, at concentrations at or below 0.1 mM the metals used to challenge Group 2 subjects with hip arthroplasty for metal reactivity (i.e. Cobalt, Chromium and Nickel) did not produce significant changes in lymphocyte proliferation in Group 1 controls when compared to unchallenged Group 1 lymphocytes.

**Figure 2:**
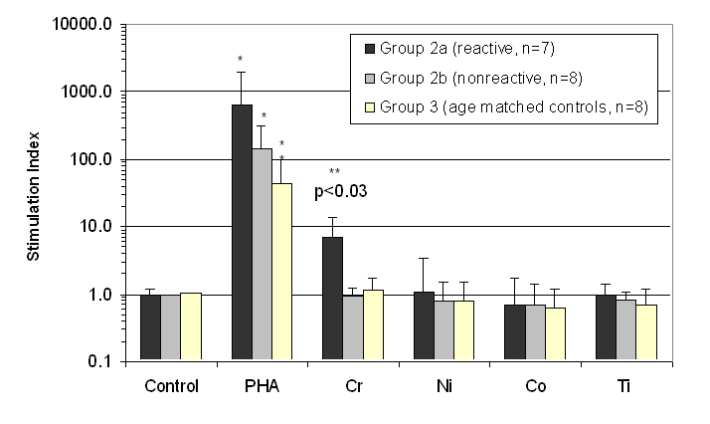
**Normalized lymphocyte proliferation responses of Group 2a and 2b hip arthroplasty subjects and Group 3 age-matched controls. **Groups 2a and 2b demonstrate statistically significant increases in lymphocyte proliferation in response to PHA relative to non-stimulated controls. Group 2a (metal-reactive) subjects demonstrated a statistically significant increase (>5 fold) in lymphocyte proliferation in response to Chromium than did Group 2b subjects (non-reactive). Note * = p < 0.03 (PHA and all other challenge conditions) and ** = p < 0.03, t-test compared to all other metal and group values.

Of the n = 15 Group 2 metal-challenged subjects with total hip arthroplasty, n = 7 demonstrated a proliferative response to 0.1 mM Chromium, Nickel, and/or Cobalt as defined by a >2 SI (p < 0.05, t-test), where 3 of 5 Group 2 subjects with moderate osteolysis were among these 7 reactive subjects. Thus elevated metal reactivity was not limited to hip arthroplasty subjects with osteolysis. None of the subjects demonstrated a reactivity (SI>2) response to 0.001 mM Titanium. These 7 reactive subjects (2 male, 5 female) were designated Group 2a (metal-reactive), while the n = 8 remaining subjects (5 male, 3 female) that did not demonstrate metal induced proliferative responses were designated Group 2b (metal nonreactive) where 2 of 8 had moderate osteolysis, as defined previously. The averaged SI reactivity for each group is shown in Fig 2. Of the 7 metal-reactive Group 2a subjects, 5 were reactive to Chromium, 1 was reactive to Nickel and Cobalt, 2 were reactive to Cobalt only and none were reactive to Titanium. The averaged responses of Group 2a demonstrated that Chromium was the only metal treatment to induce significant increases in proliferation when compared to untreated controls. Groups 1, 2a, 2b and 3 demonstrated appropriate proliferation responses to the positive control, PHA, (SI>10).

### Cytokine Analysis

**Figure 3:**
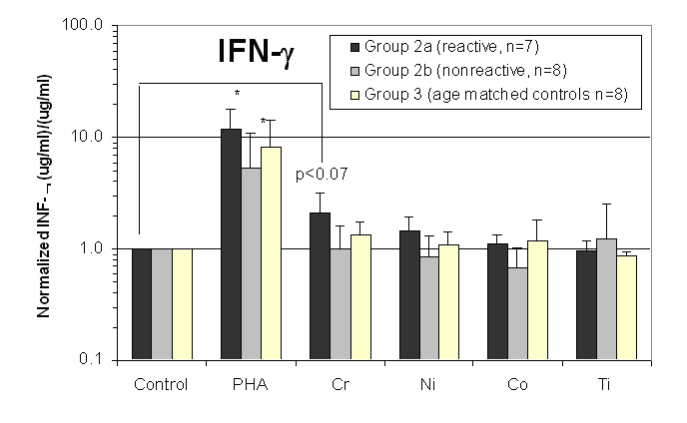
**The graphical results show the averaged (and individual normalized) IFN-gamma cytokine response of Group 2a and 2b hip arthroplasty subjects lymphocytes and Group 3 age matched controls.** Groups 2a and 2b demonstrate statistically significant increases in IFN-gamma secretion in response to PHA compared to untreated and metal challenged lymphocytes. Group 2a (metal-reactive) subjects demonstrated a statistically significant increase (>2 fold) of IFN-gamma secretion in response to Chromium than did Group 2b subjects (non-reactive) or untreated control lymphocytes of the same group. Note * = p < 0.05, t-test.

**Figure 4:**
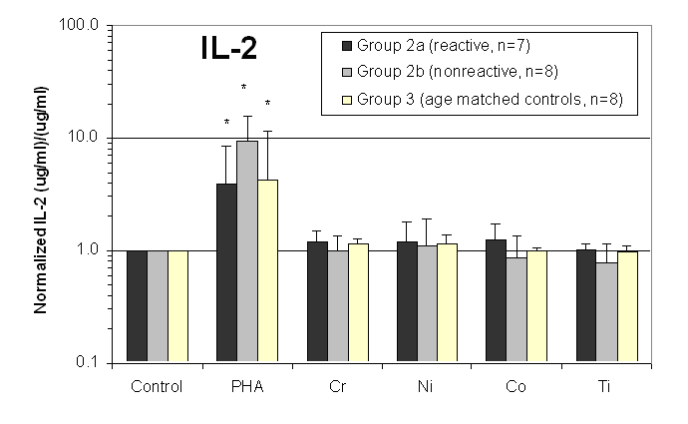
**The graphical results show the averaged (and individual normalized) IL-2 cytokine response of Group 2a and 2b hip arthroplasty subjects Group 3 age matched controls.** Groups 2a and 2b demonstrate statistically significant increases in IL-2 secretion in response to PHA compared to untreated and metal challenged lymphocytes. Group 2a (metal-reactive) subjects demonstrated a statistically significant increase (>2 fold) of IL-2 secretion in response to Chromium than did Group 2b subjects (non-reactive) or untreated control lymphocytes of the same group. Note * = p < 0.05, t-test.

**Figure 5:**
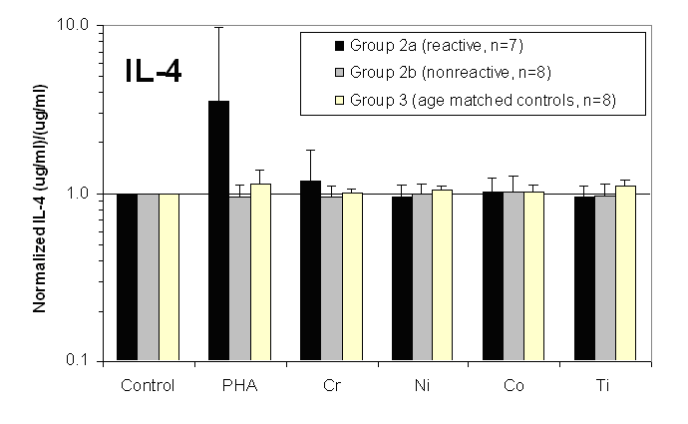
**The graphical results show the averaged (and individual normalized) IL-4 cytokine response of Group 2a and 2b hip arthroplasty subjects and Group 3 age matched controls.** Groups 2a and 2b did not demonstrate statistically significant increases of IL-4 secretion in response to PHA compared to untreated and metal challenged lymphocytes. Likewise, metal challenge did not result in increases in IL-4 secretion.

All 15 Group 2 subjects' lymphocytes were exposed to metal and the cell culture supernatant was evaluated for IFN-gamma, IL-2, and IL-4 cytokine release. The amount of cytokine release was normalized to the individual (unchallenged cells from the same individual) and averaged. These normalized amounts of IFN-gamma, IL-2, and IL-4 cytokine release for Groups 2a, 2b and 3 are shown in Figs. 3, 4, 5. Significantly elevated levels of IFN-gamma were produced in response to Chromium for Group 2a (metal-reactive) subjects (Figs. 3 and 4). IL-4 levels were not elevated in response to metal treatments in Group 2a, 2b or 3 (Fig 3). Group 2b subjects did not exhibit statistically elevated levels of IFN-gamma, IL-2, or IL-4 to metal treatment with Chromium, Nickel, Cobalt or Titanium. Proliferation and production of IFN-gamma and IL-2 in response to PHA were significantly elevated above non-challenged lymphocytes for each individual. IL-4 was not elevated after stimulation with PHA for Groups 2a, 2b and 3. Although average IL-4 release to PHA was 3-fold greater in Group 2a than in Group 2b, this increase was not statistically significant due to the variability of responses within Group 2a. Normalization to proliferation (i.e. cell number) produced non-statistically different group differences for IFN-gamma, IL-2 and IL-4. This type of double normalization was not deemed appropriate here because of the toxic effects metal challenge agents had on some individuals, where 10 of 15 Group 2 subjects demonstrated SI<0.5 (toxicity) to one or more of the metal challenge agents indicative of toxicity. This produces artificially high background variability of basal levels of cytokine, which are amplified through non-standard double normalization (proliferation and cytokine) analysis.

## Discussion

The detection of in increase in proliferation and IFN-gamma in the absence of detectable IL-4 in the PBMCs of metal-reactive hip arthroplasty patients (SI>2) supports our hypothesis that Th1 type reactivity may dominate lymphocyte reactivity responses to metals in patients with TJRs. This finding helps to identify the cellular pathways in metal-induced reactivity responses to orthopaedic implants. Typically, the principle function of Th1 cells is to aid other leukocytes, i.e. macrophages and natural killer (NK) cells, in the protective response against intracellular microbes.

Lymphocyte metal-reactivity reported here using controls (primary human lymphocytes from 8 healthy individuals with no implants and no history of metal allergy revealed that more reactive metals induce greater lymphocyte reactivity at higher concentrations tested, i.e. Nickel and Chromium (0.05–0.5 mM). Other metals (i.e. Manganese, Copper, and Vanadium) induced cell toxicity at concentrations where other metal ions (Nickel) induced a proliferative response (as low as 0.05 mM). However, metal challenge at 0.1 mM demonstrated no toxicity or statistically elevated reactivity in Group 1 normal controls (Fig 1) and was thus used as an appropriately high dose for Group 2 subjects with implants. These results of Group 1 controls are consistent with past patch-test investigations where Nickel has shown the highest prevalence of metal reactivity among the general population at an approximately 14% incidence. [1] Roughly 2 of 8 (25%) of the Group 1 young healthy controls without implants demonstrated metal reactivity to Cobalt, Chromium, or Nickel (at 0.1 mM) compared to 7 of 15 (46%) of the Group 2 subjects with well functioning hip arthroplasties (SI>2, p < 0.05 t-test) and 1 of 8 (12%) of the Group 3 age matched controls. This incidence of metal reactivity in subjects with implants (Group 2) is higher than that of the general population with total joint arthroplasty, yet lower than a previously reported 60% incidence associated with failing TJAs (prior to revision). [25-27] These results support previous reports indicating greater sensitivity associated with lymphocyte transformation testing than dermal patch testing. [28-33]

The subjects with well performing hip arthroplasties demonstrated a higher incidence and greater degree of metal reactivity, as determined by lymphocyte transformation testing to chromium, than healthy controls. This may indicate a sensitizing effect of hip arthroplasty on peripheral lymphocytes. Although the relationship between lymphocyte transformation testing and clinical TJA outcome remains undetermined, these results are consistent with the theory of individual dependent generalized idiopathic gradual sensitization to metal debris from implant degradation. The clinical significance of these findings is unknown, in part, because it is not currently feasible to compare implant performance in prospective groups with and without metal reactivity. Continued efforts to follow-up and correlate implant performance with lymphocyte reactivity will ultimately help determine the clinical utility and diagnostic capability of such assays.

These reactivity results are novel in that direct cytokine evidence from metal-stimulated PBMCs of individuals with hip arthroplasties supports the hypothesis that a Th1 activation type paradigm is identifiable in as-tested metal-reactive subjects with TJA. This Th1 reactivity may be important to the pathogenesis of aseptic osteolysis. [34,35] While activated osteoclasts are considered to be the effector cell type in bone resorption, all cell types within the periprosthetic space can contribute to the osteolytic cascade. Particles phagocytosed by macrophages and metal-protein complexes interacting with lymphocytes can lead to the production of factors, which act in both an autocrine and paracrine fashion to contribute to either increased bone resorption or reduced bone formation. IFN-gamma is produced by Th1, cytotoxic T-cells and Natural Killer T-cells and plays a pivotal role in the regulation of immune responses. IFN-gamma has been previously reported to be a potent inhibitor of osteoblast functions directly [36-38] and has been established as the predominant macrophage activating factor by priming or stimulating the expression of both class I and class II MHC (major histocompatibility complex) molecules, additional co-stimulatory macrophage cytokines (e.g. IL-1, IL-6 and TNF-α) and surface molecules (e.g. ICAM-1, B7, and CD40). [39-42]

**Figure 6:**
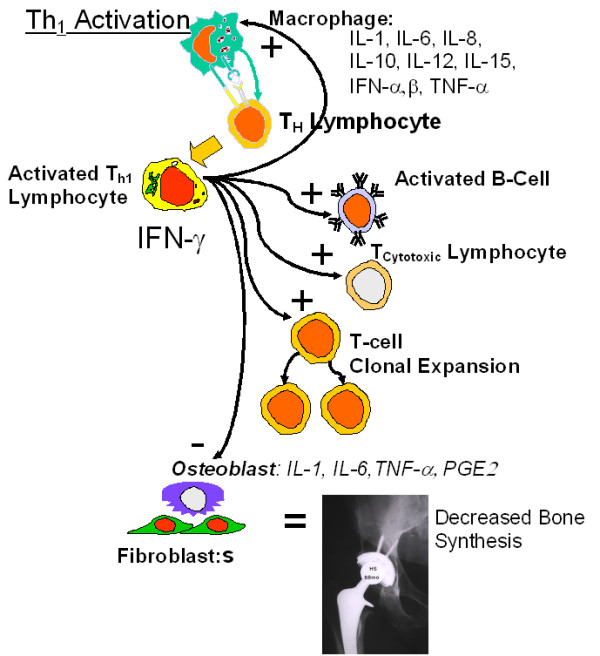
Schematic diagram of the inflammatory and peri-implant cytokine release cascade associated with activation of Th1 lymphocytes, and the inhibitory effects on bone function.

When combined, the release of cytokines IFN-gamma and IL-2 by lymphocytes can induce macrophage production of TNF-α which can establish an autocrine stimulatory loop required for continued macrophage activation and recruitment. [43-47] Therefore the present study demonstrates likely mechanisms by which lymphocyte mediated metal reactivity may contribute to the etiology of macrophage- and particle-induced osteolysis (Figure 6). IFN-gamma released by metal-activated Th1 lymphocytes is also important to macrophage activation because it is necessary for TNF-alpha released from macrophages to synergize with IFN-gamma in an autocrine fashion to induce a variety of macrophage activation genes including nitric oxide synthase, also implicated in aseptic osteolysis. [37,48] Therefore, it may be less important that a Th1 or Th2 paradigm has been determined by cytokine profiling and more important that certain cytokines themselves have been identified especially in the context of recent reports where the identification of discrete T-helper cell populations seem to be constantly updated and revised. [23]

**Figure 7:**
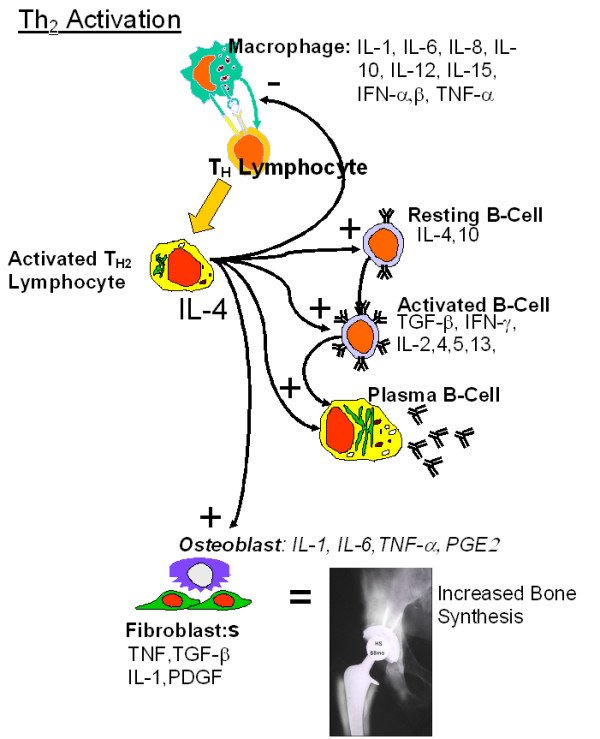
Schematic diagram of the inflammatory and peri-implant cytokine release cascade associated with activation of Th2 lymphocytes and the stimulatory effects on bone function.

IL-4, in the presence of activated T-cells, is able to trigger an IL-4-dependent activation of antibody secreting B-cells from resting B-cell populations. The implications of the lack of IL-4 secretion from lymphocytes in Group 2a and 2b subjects is that B-cell reactivity is not dominantly stimulated in these in vitro elicited metal responses (Figure 7). [42,49] Th2 cytokines such as IL-4 and IL-10 have been shown to have very powerful inhibitory effects on nearly every facet of macrophage function. Both IL-4 and IL-10 inhibit the production of such inflammatory cytokines as IL-6 and TNF-alpha [43,47] and the generation of reactive oxygen intermediates. [42,50,51] Given that the osteolysis-inducing effects of IFN-gamma released from Th1 lymphocytes are in direct contrast to the bone formation effects of released from Th2 T-cells (Figs 6 and 7), if metal reactivity responses of hip arthroplasty patients resulted in IL-4 release from Th2 cells, the ramifications to peri-implant bone homeostasis would be vastly different than the Th1-derived IFN-gamma release detected in this investigation. [24,43,47,52]

Peri-implant lymphocyte response of an individual to metal challenge remains a complex process, which can only be approximated in vitro. Our findings of IFN-gamma release in the absence of IL-4 secretion in those subjects with hip arthroplasty that demonstrated metal-reactivity (SI>2, p < 0.05) support a Th1-type reactivity paradigm. The involvement of a specific lymphocyte subtype (Th1) in the metal reactivity response implicates an *adaptive *immunity response, which represents a departure from the *innate-only *immunity response (nonspecific) typically associated with implant degradation products. It remains uncertain to what degree lymphocyte metal-reactivity contributes to the pathogenesis of poor implant performance. Continued clinical correlation between metal reactivity and implant performance (with LTT-like assays) as well as further investigation into basic mechanisms of metal antigenicity are required to build a more certain etiological connection.

## Abbreviations

Al (Aluminum), B7 (T-cell co-stimulatory molecule B7), CD4+Alpha-Beta-TCR (T-cell receptor of the type alpha beta), CD40 (T-cell co-stimulatory molecule CD40), Co (Cobalt), CoCl2 (Cobalt Chloride), Cr (Chromium), CrCl3 (Chromium Chloride), Cu (Copper), DTH, (Delayed Type Hypersensitivity), ELISA, (Enzyme Linked Immunosorbent Assay), Fe (Iron), Icam-1 (Adhesion molecule Icam-1 in leukocytes and endothelial cells), IFN-gamma, (Interferon Gamma), IL4 (Interleukin-4), IL6, (Interleukin-6), Mn (Manganese), Mo (Molybdenum), Na (Sodium), Ni (Nickel), NiCl2 (Nickel Chloride), PBMC (Peripheral Blood Mononuclear Cells), PHA (Phytohemagglutinin), Tc (Cytotoxic T cell), Th1 (Type 1 T Helper cell), Th2 (Type 2 T Helper cell 2), THA (Total Hip Arthroplasty), TJA (Total Joint Arthroplasty), TNF-alpha (Tumor Necrosis Factor Alpha), UHMWPE (Ultra High Molecular Weight Polyethylene), V (Vanadium)

## Competing interests

The author(s) declare that they have no competing interests.

## Authors' contributions

NJH designed the investigation protocol, assisted in all laboratory testing and data acquisition and prepared the manuscript. MSC assisted with all laboratory testing and data acquisition. JJJ and AF assisted with the investigation concept and design, and with manuscript preparation. All authors have read and approved the final manuscript.
